# Diastolic dysfunction in a pre-clinical model of diabetes is associated with changes in the cardiac non-myocyte cellular composition

**DOI:** 10.1186/s12933-021-01303-9

**Published:** 2021-06-01

**Authors:** Charles D. Cohen, Miles J. De Blasio, Man K. S. Lee, Gabriella E. Farrugia, Darnel Prakoso, Crisdion Krstevski, Minh Deo, Daniel G. Donner, Helen Kiriazis, Michelle C. Flynn, Taylah L. Gaynor, Andrew J. Murphy, Grant R. Drummond, Alexander R. Pinto, Rebecca H. Ritchie

**Affiliations:** 1 Heart Failure Pharmacology, Drug Discovery Biology, Monash Institute of Pharmaceutical Sciences, 399 Royal Parade, Parkville, VIC 3052 Australia; 2 Cardiac Cellular Systems, Baker Heart and Diabetes Institute, 75 Commercial Road, Melbourne, VIC 3004 Australia; 3 Haematopoiesis and Leukocyte Biology, Baker Heart and Diabetes Institute, Prahran, VIC Australia; 4 Preclinical Cardiology, Microsurgery & Imaging Platform, Baker Heart and Diabetes Institute, Prahran, VIC Australia; 5 Department of Physiology, Microbiology and Anatomy, La Trobe University, Bundoora, VIC Australia; 6 Baker Department of Cardiometabolic Health, The University of Melbourne, Parkville, VIC 3010 Australia

**Keywords:** Cardiac cellularity, Diabetes, Flow cytometry, Echocardiography, Fibroblast

## Abstract

**Background:**

Diabetes is associated with a significantly elevated risk of cardiovascular disease and its specific pathophysiology remains unclear. Recent studies have changed our understanding of cardiac cellularity, with cellular changes accompanying diabetes yet to be examined in detail. This study aims to characterise the changes in the cardiac cellular landscape in murine diabetes to identify potential cellular protagonists in the diabetic heart.

**Methods:**

Diabetes was induced in male FVB/N mice by low-dose streptozotocin and a high-fat diet for 26-weeks. Cardiac function was measured by echocardiography at endpoint. Flow cytometry was performed on cardiac ventricles as well as blood, spleen, and bone-marrow at endpoint from non-diabetic and diabetic mice. To validate flow cytometry results, immunofluorescence staining was conducted on left-ventricles of age-matched mice.

**Results:**

Mice with diabetes exhibited hyperglycaemia and impaired glucose tolerance at endpoint. Echocardiography revealed reduced E:A and e’:a’ ratios in diabetic mice indicating diastolic dysfunction. Systolic function was not different between the experimental groups. Detailed examination of cardiac cellularity found resident mesenchymal cells (RMCs) were elevated as a result of diabetes, due to a marked increase in cardiac fibroblasts, while smooth muscle cells were reduced in proportion. Moreover, we found increased levels of Ly6C^hi^ monocytes in both the heart and in the blood. Consistent with this, the proportion of bone-marrow haematopoietic stem cells were increased in diabetic mice.

**Conclusions:**

Murine diabetes results in distinct changes in cardiac cellularity. These changes—in particular increased levels of fibroblasts—offer a framework for understanding how cardiac cellularity changes in diabetes. The results also point to new cellular mechanisms in this context, which may further aid in development of pharmacotherapies to allay the progression of cardiomyopathy associated with diabetes.

**Supplementary Information:**

The online version contains supplementary material available at 10.1186/s12933-021-01303-9.

## Background

Diabetes mellitus is a leading cause of death worldwide, with a total global prevalence exceeding 450 million individuals [1]. In 2015, diabetes was attributed to 12.8% of total all-cause mortality worldwide, providing a substantial socioeconomic burden and health concern [2, 3].

Diabetes is associated with a significantly elevated risk of cardiovascular death and hospitalisation for heart failure (HF) [4, 5]. However, there remains no specific treatment for HF or its development in individuals with diabetes. HF in diabetes is often accompanied by impaired cardiac output, cardiac fibrosis, cardiomyocyte hypertrophy, cell death, and oxidative stress [6]. Diabetes also involves chronic and systemic inflammation [7, 8] with monocytosis and neutrophilia [7–9]. Despite extensive efforts to characterise diabetes-induced HF, inherent cellular mechanisms underpinning cardiac dysfunction in diabetes remain to be ascertained.

The mammalian heart consists of a diverse range of cell types [10]. Cardiac non-myocytes—comprised of endothelial cells (ECs), resident mesenchymal cells (RMCs) and leukocytes—outnumber myocytes, and are critical for maintaining homeostasis of the heart [10, 11]. While a number of recent studies have provided valuable new insights into the disparate roles of non-myocyte cells in cardiac homeostasis [10, 12, 13] and pathological remodelling [14–16], the cellular dynamics of non-myocytes during development of diabetes-induced heart failure remains unexplored. Using a recently published murine model of diabetes-induced cardiomyopathy [17], this study aimed to determine the difference in cardiac non-myocyte cellular proportions compared to non-diabetic mice. Here, we show that experimental diabetes impacts multiple cellular compartments in the heart, providing a framework for understanding the cellular dynamics and mechanisms driving development of diabetes-induced heart failure.

## Research design and methods

### Animal experiments

All animal-related experiments were approved by the Alfred Research Alliance (ARA) Animal Ethics Committee (Ethics number: E/1681/2016/B) and were performed in accordance with the National Health and Medical Research Council of Australia. FVB/N mice were sourced from the ARA Animal Services (provided in three separate cohorts). Mice had access to food and water ad libitum and were housed at 22 °C on a 12 h light/dark cycle. Male 6-week-old FVB/N mice were randomly allocated into the non-diabetic (ND, n = 7) citrate vehicle control group fed standard chow diet, or diabetes mellitus (diabetes, n = 19) which was induced by the combination of low-dose streptozotocin (STZ; cat# AG-CN2-0046, AdipoGen Life Sciences, NSW, Australia) and high-fat-diet (HFD; SF04-001, Specialty Feeds, WA, Australia, 43% total calculated digestible energy from lipids). STZ was administered by three consecutive daily intraperitoneal (i.p.) injections (55 mg/kg body weight in 0.1 mol/L citric acid vehicle, pH 4.5 [cat# 251275, Sigma-Aldrich, USA]). Mice administered STZ were subsequently fed a HFD ad libitum for 26-weeks, as previously described [17]. Blood glucose levels were measured fortnightly via saphenous vein bleeds using a glucometer (Accu-Chek® Performa II, Roche Diagnostics, NSW, Australia). Intraperitoneal glucose and insulin tolerance tests were conducted at endpoint (26-weeks of diabetes) to assess glucose clearance and insulin resistance, as previously described [17]. Whole-body composition analysis was performed at endpoint using an Echo-MRI™ 4-in-1 700 Analyser (EchoMRI, Houston, TX, USA) to assess percentage fat mass and total lean mass. Percentage glycated haemoglobin (% HbA_1c_) was also measured at endpoint to assess long-term blood glucose levels (Cobas b 101 POC system, Roche Diagnostics, NSW, Australia). Mice were euthanised by administration of Ketamine/Xylazine (85/8.5 mg/kg, i.p.) and subsequent cardiac exsanguination. As previously described [10–12], the thoracic cavity was exposed and right atrium was cut to allow for cardiac perfusion through the left-ventricular apex (PBS, 0.9 mM CaCl_2_, 200 mM KCl), after which the heart was excised and ventricles were used for flow cytometry.

### Echocardiography

Echocardiography was conducted in mice under anaesthesia (Ketamine/Xylazine/Atropine [KXA], 80/8/0.96 mg/kg, i.p.) at 26-weeks post diabetes (32-weeks of age) using a Philips iE33 ultrasound machine with a 15-MHz linear-array transducer. Analysis was conducted at the Baker Heart and Diabetes Institute and quality control was completed by technicians at the Preclinical Cardiology Microsurgery & Imaging Platform (PCMIP). Doppler flow echocardiography was used to assess cardiac transmitral flow velocity in each phase of diastole, where the early phase (E wave) and the late phase (A wave) were measured to determine the E:A ratio. Similarly, tissue Doppler was performed to examine the tissue motion of the mitral annulus (early phase = e’, late phase = a’ wave). M-mode echocardiography was conducted to assess left ventricle (LV) systolic function. Variables obtained from M-mode analysis included LV end-diastolic dimension (LVEDD) and LV end-systolic dimension (LVESD) to calculate fractional shortening (%FS = [(LVEDD-LVESD)/LVEDD] × 100).

### Flow cytometry

#### Blood, spleen and bone marrow

Whole blood was obtained by cardiac puncture at endpoint and stained using a leukocyte-specific antibody panel (Additional file 1: Table S4). Bone marrow from the tibia and femur were flushed using PBS without Mg^2+^ and Ca^2+^ into 50 mL centrifuge tubes. Spleens were manually dissociated and passed through a 35 µM filter into 50 mL centrifuge tubes to obtain a single cell suspension as previously described [7]. Blood, spleen and bone marrow were then subjected to red blood cell (RBC) lysis for 15 min at 4 °C using an ammonium chloride based commercial lysis buffer (1X dilution, 555899, Becton Dickinson, USA). After RBC lysis, the remaining stained cells were washed twice in ‘Fx buffer’ (1 X HBSS [Gibco™, NY, USA], 2% FCS). Between each wash, cells were centrifuged at 400 × *g* for 5 min at 4 °C. Cells were then resuspended in 200 µl of Fx buffer containing 4′,6-diamidino-2-phenylindole (DAPI [0.1 µg/mL]); and filtered through 35 µM mesh into 5 ml polystyrene round-bottom tubes (352052, Falcon®, NY, USA) for flow cytometry. Gating strategies for each of the above cell suspensions are provided in Additional file 1: Figures S3–S5. For normalisation of flow cytometry data, 20 µl of blood was used to measure total white blood cell count using a Sysmex XS-1000i Hematology Analyzer.

### Heart

High-dimensional flow-cytometry was performed on cardiac ventricles (comprising the LV, ventricular septum and right ventricle) from ND and mice with diabetes. Following perfusion, hearts were minced using curved scissors (14077-09, Walton, USA) as previously described [10], and transferred to 5 ml microfuge tubes for enzymatic digestion at 37 °C (2 mg/mL collagenase type IV [LS004188, Worthington Biochem, NJ, USA], 1 mg/mL Dispase II [04942078001, Roche, NSW, Australia] in 0.9 mM CaCl_2_ in PBS). Cardiac non-myocyte cells were triturated three times at 15-min intervals using a Pasteur pipette to mechanically aid enzymatic digestion for a total of 45 min. Digested non-myocyte cardiac cells were then filtered through 75 µM nylon mesh into a 15 mL tube containing 10 mL of cold PBS (0.9 mM CaCl_2_) and subjected to centrifugation (200* g*, 15 min, 4 °C—no breaks) for debris clearance. The majority of the supernatant was aspirated and the remaining volume (~ 1 mL) was washed with a further 1 mL of Fx buffer supplemented with 0.9 mM CaCl_2_. Cells were pelleted at 400 × *g* (4 min, 4 °C) and resuspended in 200 µl of Fx Buffer with Ca^2+^ to yield the single cell suspension of non-myocyte cardiac cells. Cells were then stained using the antibody panel designed for examining the non-myocyte fraction of the heart (Additional file 1: Table S5). Cells were strained through a 35 µm filter and flow cytometry was performed on a BD LSR Fortessa™ X-20 Special Order system located at the Baker Heart and Diabetes Institute.

### Histological analysis

Age and sex-matched, fresh-frozen LV samples embedded in Optimal Cutting Temperature (OCT) compound were acquired from a separate cohort of ND and mice with diabetes [17] for histological analysis. LV sections were cut (10 µm) on a cryostat (CM1950, Leica Biosystems) for staining (ND: *n* = 11, diabetes: *n* = 11). LV sections were co-stained with GATA4 (1:100, 14-9980-80, eBioScience™, Invitrogen, Australia) and PCM1 (1:100, 19856-1-AP, ProteinTech Group, USA) antibodies to delineate the cell abundance of RMCs (PCM1^−^GATA4^+^ cells) as recently reported [12]. Serial sections were stained with DACH1 (1:100, 10914-1-AP, ProteinTech, USA) to quantify EC abundance [10, 12]. All immunofluorescence sections were counterstained with DAPI to identify total cell nuclei. Immunofluorescence micrographs of each LV sample were acquired at a 20X objective and tiled (3 × 3 fields of view) on a Nikon A1R confocal laser scanning microscope. Quantified values of immunofluorescence signal were normalised to total nuclei (DAPI^+^).

### Statistical analysis

Flow cytometry data was analysed using FlowJo (v10.7.1) software. Raw cardiac flow cytometry data was normalised to the mean of the ND values within each batch, such that the mean of each ND cell type is equal to 1. Raw blood flow cytometry data was normalised to total white-blood cell count obtained from the hematology analyser, then subsequently batch normalised as aforementioned. Immunofluorescence micrographs were analysed by QuPath software (v0.2.3), using the cell count function to quantify nuclei. Echocardiography data was analysed using RadiAnt DICOM viewer software (v2020.2), after which quantification was performed in accordance with the PCMIP guidelines. All data was illustrated and analysed statistically using GraphPad Prism (v8.1.2). Comparison of experimental groups was conducted using an unpaired *t*-test, whereby statistical significance was determined as *P* < 0.05.

## Results

### The STZ-HFD model recapitulates primary features of diabetes

The presence of diabetes was confirmed by a range of physiological tests prior to euthanasia. Consistent with our previous report [17], mice with diabetes exhibited significantly elevated blood glucose at endpoint (Additional file 1: Table S1). This was corroborated by measurement of glycated haemoglobin (% HbA_1c_) at endpoint, which was significantly increased in mice with diabetes (*P* < 0.0001; Additional file 1: Table S1). In this study however, mice exhibiting diabetes did not gain more weight than their ND counterparts (Additional file 1: Table S1). This was recapitulated by the EchoMRI body composition analysis, showing no differences in lean or fat mass (Additional file 1: Table S1) between experimental groups. Impaired glucose tolerance was evident in mice with diabetes, indicating reduced clearing efficiency of systemic glucose, presented as the area under the curve (AUC, *P* < 0.0001, Additional file 1: Table S1). In contrast, there was no difference in the AUC from the insulin tolerance test between ND and mice with diabetes (Additional file 1: Table S1).

### STZ-HFD mice exhibit LV diastolic dysfunction, but not systolic dysfunction

Echocardiography measurements of LV diastolic and systolic function were recorded in vivo, to determine the degree of cardiac functional impairment in mice with diabetes relative to their ND counterparts. Pulsed-wave Doppler echocardiography was conducted to measure mitral blood flow velocity during the early (E-wave) and late (A-wave) filling phases of diastole (Additional file 1: Fig. S1A). Heart rate (HR) tended to be elevated in mice with diabetes, but this did not reach statistical significance (*P* = 0.07; Additional file 1: Figure S1B). Although no differences were detected in the peak E wave (Additional file 1: Figure S1C), the peak A wave velocity was significantly elevated in mice with diabetes compared to ND mice (*P* < 0.05; Additional file 1: Figure S1D). Consequently, a significant reduction in E:A ratio (a hallmark feature of diastolic dysfunction) was observed in diabetic hearts vs. ND (*P* < 0.05 Additional file 1: Figure S1E). There were no differences in other measurements of diastolic function including deceleration time or isovolumic relaxation time (IVRT) between experimental groups (Additional file 1: Figure S1F, G, respectively).

To accompany transmitral blood flow, tissue Doppler echocardiography was used to assess the velocity of the mitral valve itself in each phase of diastole (e’ = early phase, a’ late phase, Additional file 1: Figure S1H–L). Although the peak e’ velocity was only modestly reduced (*P* = 0.054, Additional file 1: Figure S1I) and the peak a’ velocity exhibited a minor increase (*P* = 0.072, Additional file 1: Figure S1J), the e’:a’ ratio was significantly lower in mice with diabetes compared to ND mice (*P* < 0.05, Additional file 1: Figure S1K). There were no detectable changes in the E:e’ ratio between cohorts (Additional file 1: Figure S1L).

M-Mode echocardiography was also performed to assess the difference in ventricular wall thickness and systolic function in mice with diabetes. The anterior wall thickness at diastole (AWd), LV end-diastolic dimension (LVEDD) and posterior wall thickness at diastole (PWd) were not different between groups (Additional file 1: Table S2). Interestingly, fractional shortening (% FS) was significantly elevated in mice with diabetes compared with ND mice (*P* < 0.05; Additional file 1: Table S2), consistent with a recent report in spontaneously type-1 diabetic (T1D) Akita mice [18]. Importantly however, diastolic dysfunction was observed in the absence of systolic dysfunction.

### Diabetes alters the cardiac non-myocyte cellular composition

**Fig. 1:**
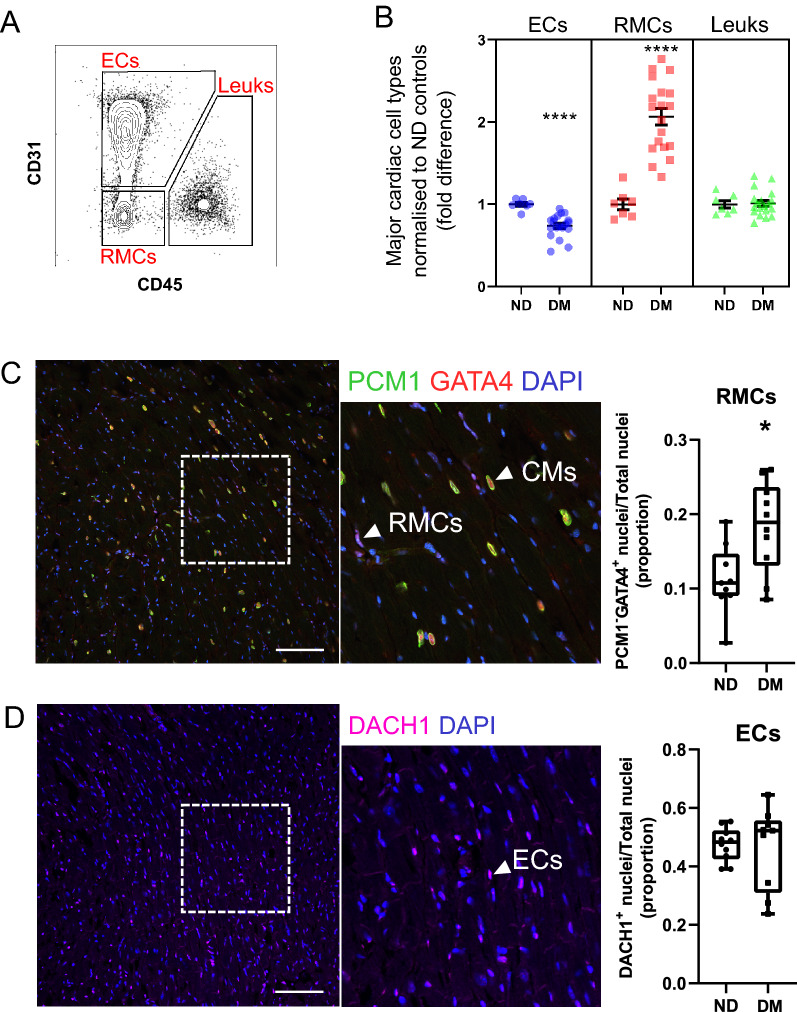
Differences in the abundance of major non-myocyte cell classes in the diabetic heart. **A** Flow cytometry contour plot displaying gating of major non-myocyte cell types for quantification of cell type proportion (summarised in B). For full gating strategy see Additional file 1: Figure S2. Endothelial cells (ECs; CD31^+^), resident mesenchymal cells (RMCs; CD31^−^CD45^−^) and leukocytes (Leuks; CD45^+^). **B** Proportions of major cell types in non-diabetic (ND; n = 7) and diabetic (DM; n = 19) mouse hearts. Individual sample values are shown with mean ± SEM. **C** Immunohistochemical analysis of the abundance of RMCs in ND and diabetic mouse heart left ventricles. Left and middle panels show representative confocal micrographs of mouse heart tissue stained for PCM1 and GATA4. PCM1^+^GATA4^+^ and PCM1^−^GATA4^+^ nuclei correspond to nuclei of cardiomyocytes (CM) and RMCs respectively. Nuclei are counterstained with DAPI. Right panel (box-plot) summarises proportion of nuclei corresponding to RMCs in ND (n = 9) vs. DM (n = 10) enumerated from micrographs. Whiskers of box-and-whisker plot indicate max and min. **(D)** As for C, heart left ventricle sections were stained with DACH1 to identify nuclei corresponding to endothelial cells in ND (n = 10) and DM (n = 9) left ventricles. *P < 0.05, **** *P* < 0.0001 (Student’s unpaired *t*-test). Scale bar = 100 µM

To assess differences in cardiac cellularity associated with diabetes-induced HF, we performed flow cytometric analysis of murine cardiac ventricles at study endpoint. Examination of viable single-cells (see Additional file 1: Figure S2A) revealed significant differences in endothelial cell (EC) and resident mesenchymal cell (RMC) proportions (0.26-fold decrease, twofold increase, respectively), indicating that diabetes alters the relative levels of cardiac non-myocyte cells (Fig. 1A, B). Conversely, leukocytes were at similar levels in ND and mice with diabetes (Fig. 1A, B).

Next, we sought to validate the proportional shifts in EC and RMC populations in diabetes observed by flow cytometry, with immunohistochemical analysis (Fig. 1C, D). To achieve this, we stained left ventricular sections of both cohorts with an antibody cocktail of GATA4 and PCM1 (Fig. 1C) or DACH1 (Fig. 1D), which we have previously employed to quantify proportions of RMCs and ECs [12]. These analyses revealed that RMC (PCM1^−^GATA4^+^) cell counts were significantly elevated in diabetic heart sections compared to ND counterparts (*P* < 0.05, Fig. 1C). Using the same approach for ECs, serial sections stained with DACH1 indicated no differences in EC abundance between experimental groups (Fig. 1D), suggesting that the proportional difference observed by flow cytometry is due to the increased RMCs [10].

**Fig. 2:**
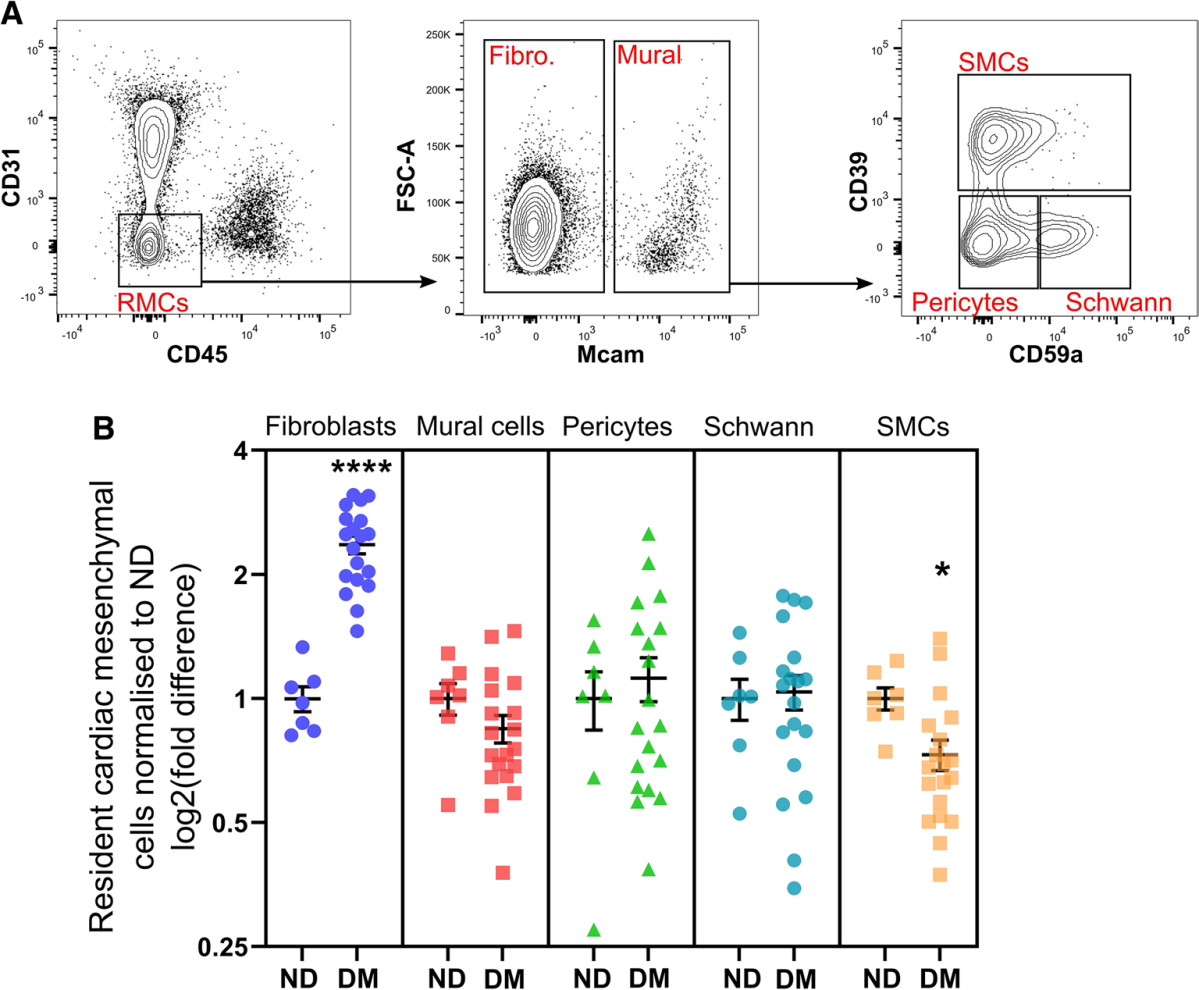
Differences in resident mesenchymal cell (RMC) subtypes in the diabetic heart. **A** Flow cytometry contour plots display gating strategy for cardiac RMCs and subsets (fibroblasts, SMCs, pericytes and Schwann cells) for quantifying RMC proportions (summarised in B). For full gating strategy see Additional file 1: Figure S2. **B** Proportions of RMC sub-classes in ND (n = 7) and diabetic (n = 19) mouse ventricles. Fibro: Fibroblast; Mural: Mural cells; SMCs: smooth muscle cells. Data is displayed as mean ± SEM. *P < 0.05, **** *P* < 0.0001 (Student’s unpaired *t*-test)

Considering the proportion of RMCs were markedly elevated in the diabetic heart, a range of RMC subtypes were investigated from the initial RMC gate (Additional file 1: Fig.S2). Fibroblasts were significantly increased in diabetic hearts compared to ND (2.36-fold, *P* < 0.0001, Fig. 2B). In contrast, the proportion of smooth muscle cells (SMCs), were reduced in the diabetic cohort compared to ND controls (0.27-fold, *P* < 0.05, Fig. 2B). No major changes were observed in total mural cells, pericyte or Schwann cell populations (Fig. 2B).

**Fig. 3:**
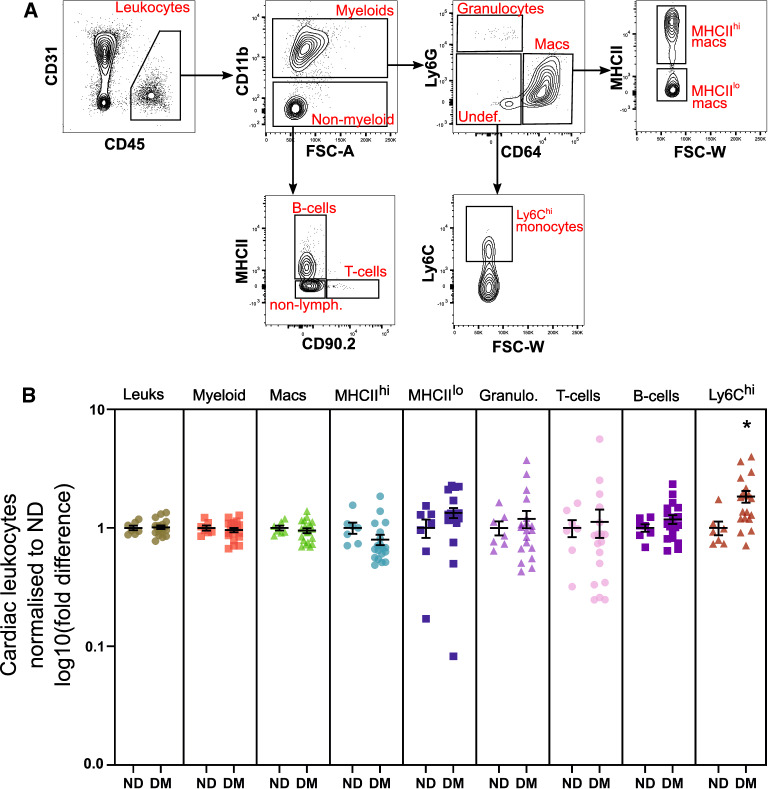
Ly6C^hi^ monocytes, but not resident leukocytes are increased in the diabetic heart. **A** Flow cytometry contour plots display gating strategy for quantifying cardiac leukocytes (summarised in B). For full gating strategy see Additional file 1: Figure S2. **B** Proportions of leukocyte sub-types in ND (n = 7) and DM (n = 19) mouse ventricles. Data is displayed as mean ± SEM. *P < 0.05 (Student’s unpaired *t*-test)

While we did not detect any changes in total resident leukocyte proportions in diabetic mouse hearts compared to ND (Fig. 1B), diabetes has been previously associated with cardiac inflammation and systemic monocytosis [7, 19, 20]. To develop an overview of leukocyte diversity and abundance in diabetic hearts, we identified an array of leukocytes including myeloid and lymphoid cell populations and their subsets (Fig. 3A). There were no differences in cardiac leukocyte subsets between cohorts, except Ly6C^hi^ monocytes, which were significantly increased in the myocardium of mice with diabetes (1.8-fold, Fig. 3B).

### Circulating Ly6C^hi^ monocytes are elevated in diabetes

**Fig. 4:**
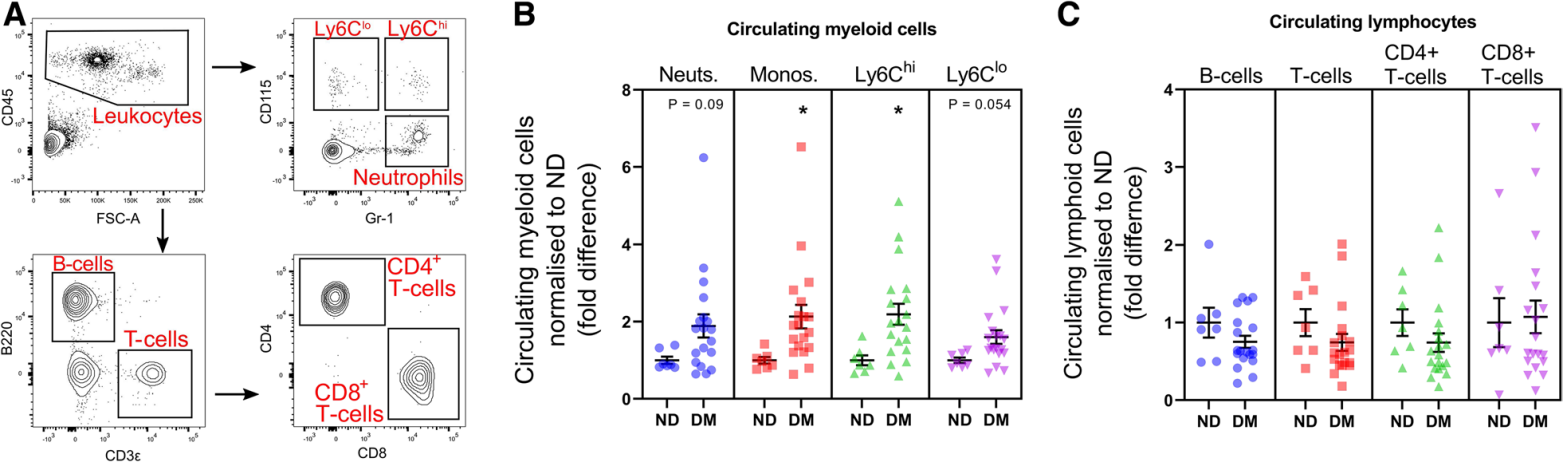
Mice with diabetes exhibit systemic monocytosis. **A** Flow cytometry contour plots display gating strategy for circulating leukocytes in whole blood (summarised in B and C). For full gating strategy see Additional file 1: Figure S2. **B–C** Proportions of circulating myeloid leukocytes and lymphocytes in ND (n = 7) vs. DM (n = 19) mice. Monocytes (Monos.), Ly6C^hi^ monocytes (Ly6C^hi^), Neutrophils (Neuts.) and Ly6C^lo^ monocytes (Ly6C^lo^). Data is displayed as mean ± SEM. *P < 0.05 (Student’s unpaired *t*-test)

To confirm systemic monocytosis, we quantified circulating leukocytes and their broad subtypes by flow cytometry. As shown previously [7], monocytes, particularly the Ly6C^hi^ subset, were significantly elevated in the blood of mice with diabetes (2.2-fold, 2.3-fold, respectively; *P* < 0.05 for both; Fig. 4B). Numbers of circulating neutrophils and Ly6C^lo^ monocytes were also marginally elevated in diabetic mice compared to their ND counterparts (*P* = 0.09, *P* = 0.054 respectively; Fig. 4B,). By contrast, numbers of circulating lymphocytes (B and T-cells) did not differ between cohorts (Fig. 4C).

### Systemic monocytosis likely occurs via extramedullary myelopoiesis

**Fig. 5:**
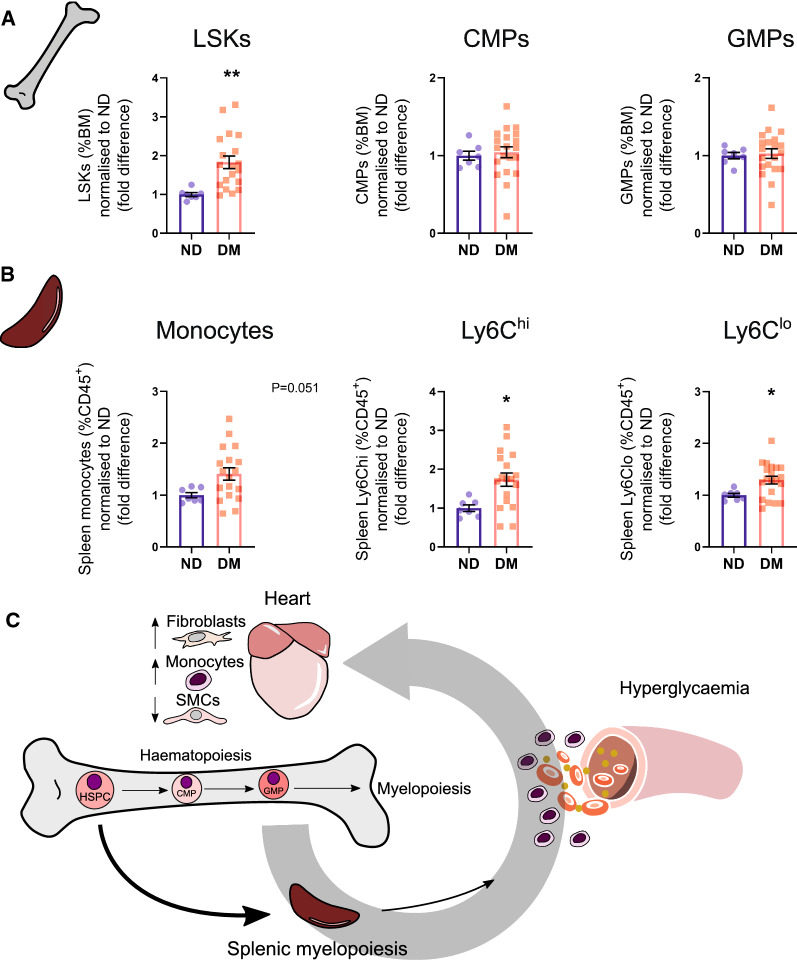
Bone marrow and spleen myelopoiesis evident in mice with diabetes. **A** Quantified proportions of bone marrow progenitor cells. LSK cells (Lineage^−^, Sca-1^+^, cKit^+^; haematopoietic stem cells), common myeloid progenitors (CMPs) and granulocyte myeloid progenitors (GMPs). **B** Proportions of spleen monocytes in ND (n = 7) vs. DM (n = 19) mice. **C** Proposed mechanism by which systemic monocytosis occurs in diabetic mice administered STZ-HFD. See Additional file 1: Figure S4 and S5 for full gating strategies for flow cytometry analysis. Data displayed as mean ± SEM. *P < 0.05, **P < 0.01 (Student’s unpaired *t*-test)

To identify the potential sources of the observed monocytosis in this model, we performed flow cytometry of the bone marrow and spleen. Within the bone marrow, LSKs (haematopoietic stem and progenitor cells; [Lin^−^Sca1^+^cKit^+^]) were significantly increased in mice with diabetes (1.8-fold, *P* < 0.01, Fig. 5A). However, bone-marrow derived common myeloid progenitors (CMP) and granulocyte-myeloid progenitors (GMP) were not different between experimental groups (Fig. 5A). Monocytes (both Ly6C^hi^ and Ly6C^lo^) were significantly increased in the spleen in mice with diabetes compared to their ND controls (1.7-fold, 1.3-fold respectively, *P* < 0.05, Fig. 5B). These data suggest that the increased proportion of bone-marrow LSKs could be influencing these cells to mobilise to the spleen to undergo extramedullary myelopoiesis (Fig. 5C).

## Discussion

The relationship between diabetes and HF remains poorly understood. Diabetes-associated cardiac remodelling— encompassing myocyte hypertrophy, fibrosis, oxidative stress and apoptosis [21] is well established. However, how the cardiac non-myocyte networks change in diabetes and contribute to this remodelling is unclear. Using a recently characterised mouse model of diabetes-induced cardiomyopathy [17], we aimed to determine how diabetes affects cardiac non-myocyte cell proportions and abundance, to develop a framework for future mechanistic studies to consider. We revealed that with diabetes-associated diastolic dysfunction, proportions of cardiac fibroblasts are significantly increased in the myocardium. We also noted increased levels of Ly6C^hi^ monocytes and decreased levels of SMCs in diabetic hearts.

Numerous studies have implicated cardiac fibroblasts in diabetic cardiomyopathy, however their precise role in diabetes in vivo is still unknown. Cardiac fibroblasts are the primary cell type involved in deposition of extracellular matrix (ECM) in both states of acute injury or chronic stress [14, 15]. However, in these contexts, fibroblast gene expression and phenotype are distinct [22]. For example, in myocardial infarction (MI), fibroblasts rapidly differentiate into activated fibroblasts and myofibroblasts—both well-established drivers of cardiac ECM deposition [23]. Conversely, we have recently reported that myofibroblasts are absent during the development of chronic fibrosis in angiotensin II-induced cardiac remodelling [14]. Observations from the present study reveal that fibroblasts are the predominant non-myocyte cell type most dramatically affected by diabetes—suggesting an important role for fibroblasts in the development of diabetes-induced HF. Indeed, chronic hyperglycaemia is known to up-regulate various pro-fibrotic genes in the diabetic heart as a whole, including *Col1a1, Postn, Timp-2* and *Ccn2* [17, 24, 25]. Furthermore, diabetes is associated with fibroblast-to-myofibroblast differentiation and ECM deposition [26, 27]. However, further research such as single-cell sequencing or targeted cell depletion experiments are needed to further elucidate the precise role of the cardiac fibroblast in diabetes and the regulatory mechanisms that drive these changes.

In the current study, we also observed an increase in monocyte numbers in the heart in diabetic mice, which is likely the result of increased systemic inflammation. In the non-injured heart, circulating leukocytes, such as monocytes primarily reside in the vascular lumens of cardiac capillaries [28], therefore reflecting changes occurring in the circulation. Systemic monocytosis is reported in both T1D and insulin resistant obese mice (i.e. leptin mutant *ob/ob* mice and diet-induced obese mice) [7, 8]. Consistent with systemic inflammation, the diabetic heart exhibits upregulation of pro-inflammatory cytokines such as TNFα, MCP-1 and IL-1β [29–31]. Corresponding to the monocytosis, we also noted increased progenitor cells and splenic monocytes—the major site of secondary myelopoiesis [32]. Monocytosis is a well-established feature of diabetes and obesity/insulin resistance [33, 34]. However, in this model we only detected a significant increase in haematopoietic stem and progenitor cells (HSPCs), but not common myeloid progenitors (CMPs) or granulocyte–macrophage progenitors (GMPs) in the bone marrow. Given HSPCs can migrate to secondary myelopoietic sites, such as the spleen, to increase monocyte numbers [35–37], our findings suggest that this may be the primary mode of monocytosis observed in our model (Fig. 5C).

In contrast to the diabetes-induced increases in cardiac fibroblasts and monocyte numbers, we observed a decrease in SMC proportions. This was unexpected given hyperglycaemia has previously been associated with inhibition of aortic vascular SMC apoptosis in T1D patients, and in STZ-induced T1D mice [38, 39]. Conversely, metabolic syndrome and hypercholesterolaemia are both associated with increased apoptosis in aortic VSMCs of mice and humans [33, 34]. Therefore, the precise mechanism leading to the reduction in SMC proportions in the hearts of STZ-HFD mice warrants further investigation.

Although there are a number of studies examining the role of individual cardiac cell types in diabetes, to our knowledge this is the first study to consider the entire cardiac non-myocyte network to understand differences in tissue cellularity. While novel technologies such as single-cell RNA sequencing have been successfully applied to tissues such as the pancreas [35, 36, 40], kidney [41, 42] and liver [43] in diabetes, detailed interrogation of the cellular heterogeneity in these tissue systems are lacking in this context. This study invites future research to consider cellular plasticity in diabetes to better understand the development of its associated pathologies.

## Study limitations

While this study provides a basis for providing new understanding of the cardiac cellular dynamics in the context of diabetes, a number of limitations are noteworthy. First, cardiomyocytes were not considered in this study, as they are too large in diameter to pass through the flow cytometer available in our laboratories. Although cardiomyocytes are detectable by histology (PCM1^+^GATA4^+^ cells), they are often multi-nucleated, thus counting nuclei abundance is unlikely to yield accurate information. Furthermore, we did not measure morphological changes in cardiomyocyte size or deposition of myocardial fibrosis, despite there being no differences in our previous characterisation of this model [17]. Second, we only examined male mice in our study. Given that cardiac pathology is sex-specific in mice [44, 45] and in humans [46], cardiac cellular composition and gene expression are sexually-dimorphic [12, 14]. Future work should examine the impact of biological sex in the development of diabetic cardiomyopathy. Third, the STZ-HFD model used in this study did not yield a population of mice with elevated fat mass and body weight as expected [47]. Adiposity and obesity are important comorbidities contributing to pathology in experimental and clinical type-2 diabetes (T2D) [48, 49], albeit obesity is not essential for development of T2D [48, 49]. Importantly however, in this study mice with diabetes exhibited hyperglycaemia, impaired glucose tolerance and LV diastolic dysfunction, which are clinically relevant features of HF associated with diabetes. Furthermore, we were unable to ascertain whether the observed differences are attributed to the combination of STZ and HFD, or one of these individual insults. Future work using this model should consider the effect of STZ and HFD alone in addition to the combination of STZ-HFD to delineate the role of both factors in the development of diabetic HF. Lastly, this study did not consider how circulating populations of cells such as mesenchymal stem cells may contribute to the cardiac RMC compartment. Future work will consider whether resident or circulating cell populations drive the expansion of fibroblasts in the diabetic heart.

## Conclusion

Here we have profiled the differences in the cardiac non-myocyte network, observing that the cellular landscape of the heart changes in a murine model of diabetes. By quantifying proportional shifts in a wide array of cell types simultaneously, these results offer a framework for understanding the cellular mechanisms that may drive pathological remodelling of the heart during the development of diabetes-induced HF. Future research will determine the precise cellular and molecular mechanisms that drive increased fibroblast numbers and the impact of this for development of diabetic cardiomyopathy. Targeting the molecular pathways that drive these non-myocyte cellular changes may offer new therapeutic avenues to address the cardiac complications associated with diabetes.

## Supplementary Information

**Additional file 1**: **Table S1**. Physiological endpoint characteristics of STZ-HFD-induced murine diabetes. **Table S2**. Endpoint M-Mode echocardiography for assessing cardiac systolic function in murine diabetes. **Table S3**. Organ weights.** Table S4**. Flow cytometry antibody panel utilised in whole blood from mice. **Table S5**. Flow cytometry antibody panel utilised in myocardium from mice. **Figure S1**. (A) Representative images of transmitral annular blood flow via Doppler echocardiography. Quantified Doppler flow; (B) Anaesthetised heart rate (HR), (C) peak E-wave velocity, (D) A-wave velocity (E) E:A ratio, (F) deceleration time (DT) and (G) isovolumic relaxation time (IVRT). (H) Shows representative images for tissue Doppler echocardiography, quantified in figures I-L. (I) Peak e' velocity (P=0.054), (J) peak a' velocity (P = 0.072), (K) e':a' ratio, (L) E:e' ratio. ND = non-diabetic, DM = diabetes mellitus. Data presented as mean ± SEM and individual data points, and analysed using an unpaired t-test. Statistical significance was assumed at P <0.05. **Figure S2**. Flow cytometry gating strategies – Heart. A) Illustrates the gating strategies used for identification of cardiac non-myocyte cell populations. Single, intact cells are first identified by the FSC-A/FSC-H gate. Next, cells are deemed ‘live and metabolically active’ by gating all SYTOX-Calcein+ events as indicated. Live, metabolically active cells were then identified based on their cell clustering to each respective antibody (listed on the x and y-axes). ECs = Endothelial cells, RMCs = Resident mesenchymal cells, Leuks = Leukocytes, Undefined = Undefined cells, Macs = Macrophages, MHCIIhi/lo = MHCIIhi/lo macrophages, Fibros = Fibroblasts, Mural = Mural cells, SMCs = Smooth Muscle Cells, Schwann = Schwann cells, FSC-A = forward scatter area, FSC-W = forward scatter width, FSC-H = forward scatter height. B) Depicts the total number of live, metabolically active cells acquired per sample (from flow cytometry), split by treatment (ND = non-diabetic, DM = diabetes mellitus. Data is presented as individual values. Each line indicates the median. P = NS (Student’s unpaired t-test). **Figure S3**. Flow cytometry gating strategies – Blood. Illustrates the gating strategies used for identification of circulating leukocyte populations. Single, intact cells are first identified by the FSC-A/FSC-H gate. Live cells were identified as DAPI- (4′,6-diamidino-2-phenylindole), after which cells are assigned as described in Supplementary Figure 2. FSC-A = forward scatter area, FSC-W = forward scatter width, FSC-H = forward scatter height. **Figure S4**. Flow cytometry gating strategies – Bone marrow. Illustrates the gating strategies used for identification of bone marrow progenitors. Lin- = lineage negative, HSPC = haematopoietic stem cell, LSK = lineage- cKit+ cells, FSC-H = forward scatter height. Lineage cocktail = CD3, CD19, CD2, B220, TER119, CD11b, Gr-1, CD8, CD4. **Figure S5**. Flow cytometry gating strategies – Spleen. Illustrates the gating strategies used for identification of splenic monocytes. SSC-A = side scatter area. **Figure S6**. Histological identification of resident mesenchymal cells. Representative micrograph of murine left-ventricle stained with PCM1 and GATA4 antibodies, counter-stained with DAPI. Monochrome images (left) indicate the positive signals acquired for nuclei enumeration. Each channel is then merged and displayed in colour (right). **Figure S7**. Histological identification of endothelial cells. Representative micrograph of murine left-ventricle stained with DACH1 and counter-stained with DAPI. Monochrome images (left) indicate the positive signals acquired for nuclei enumeration. Both channels are then merged and displayed in colour (right). **Figure S8**. Chronic hyperglycaemia is evident in diabetic mice throughout study duration. Hyperglycaemia was first detected 2-weeks after the commencement of STZ-HFD administration, and remains elevated until endpoint (measured fortnightly). Data is presented as mean ± SEM. Statistical significance was determined by a repeated measures ANOVA using a Tukey’s multiple comparison post-hoc test. **P < 0.01, ***P < 0.001, ****P < 0.0001.

## Data Availability

The datasets used and/or analysed during the current study are available from the corresponding author on reasonable request.
