# Supplementary material for: Diastolic dysfunction in a pre-clinical model of diabetes is associated with changes in the cardiac non-myocyte cellular composition
Source: Cardiovasc Diabetol. 2021 Jun 1;20:116. doi: 10.1186/s12933-021-01303-9 (PMC8170962; doi:10.1186/s12933-021-01303-9)
Supplement: Supplementary file 1 — Additional file 1: Table S1. Physiological endpoint characteristics of STZ-HFD-induced murine diabetes. Table S2. Endpoint M-Mode echocardiography for assessing cardiac systolic function in murine diabetes. Table S3. Organ weights. Table S4. Flow cytometry antibody panel utilised in whole blood from mice. Table S5. Flow cytometry antibody panel utilised in myocardium from mice. Figure S1. (A) Representative images of transmitral annular blood flow via Doppler echocardiography. Quantified Doppler flow; (B) Anaesthetised heart rate (HR), (C) peak E-wave velocity, (D) A-wave velocity (E) E:A ratio, (F) deceleration time (DT) and (G) isovolumic relaxation time (IVRT). (H) Shows representative images for tissue Doppler echocardiography, quantified in figures I-L. (I) Peak e' velocity (P=0.054), (J) peak a' velocity (P = 0.072), (K) e':a' ratio, (L) E:e' ratio. ND = non-diabetic, DM = diabetes mellitus. Data presented as mean ± SEM and individual data points, and analysed using an unpaired t-test. Statistical significance was assumed at P <0.05. Figure S2. Flow cytometry gating strategies – Heart. A) Illustrates the gating strategies used for identification of cardiac non-myocyte cell populations. Single, intact cells are first identified by the FSC-A/FSC-H gate. Next, cells are deemed ‘live and metabolically active’ by gating all SYTOX-Calcein+ events as indicated. Live, metabolically active cells were then identified based on their cell clustering to each respective antibody (listed on the x and y-axes). ECs = Endothelial cells, RMCs = Resident mesenchymal cells, Leuks = Leukocytes, Undefined = Undefined cells, Macs = Macrophages, MHCIIhi/lo = MHCIIhi/lo macrophages, Fibros = Fibroblasts, Mural = Mural cells, SMCs = Smooth Muscle Cells, Schwann = Schwann cells, FSC-A = forward scatter area, FSC-W = forward scatter width, FSC-H = forward scatter height. B) Depicts the total number of live, metabolically active cells acquired per sample (from flow cytometry), split by tre [file 12933_2021_1303_MOESM1_ESM.pdf]

Additional file 1

Diastolic Dysfunction in a Pre-Clinical Model of Diabetes is Associated with Changes in the Cardiac Non-myocyte Cellular Composition

Cardiovascular diabetology

Charles D. Cohen[1,2,5], Miles J. De Blasio[1], Man K. S. Lee[3], Gabriella E. Farrugia[2], Darnel Prakoso[1], Crisdion Krstevski[2,5], Minh Deo[1], Daniel G. Donner[4,6], Helen Kiriazis[4,6], Michelle C. Flynn[3], Taylah L. Gaynor[2,5], Andrew J. Murphy[3], Grant R. Drummond[5], Alexander R. Pinto[2,5]*, Rebecca H. Ritchie[1,5]*.

[1]Heart Failure Pharmacology, Drug Discovery Biology, Monash Institute of Pharmaceutical Sciences, Parkville, VIC; [2]Cardiac Cellular Systems, [3]Haematopoiesis and Leukocyte Biology, [4]Preclinical Cardiology, Microsurgery & Imaging Platform, Baker Heart and Diabetes Institute, Prahran, VIC; [5]Department of Physiology, Microbiology and Anatomy, La Trobe University, Bundoora, VIC, [6]Baker Department of Cardiometabolic Health, The University of Melbourne, Parkville VIC 3010, Australia.

*Equal contribution

Supplementary Table 1 – Physiological endpoint characteristics of STZ-HFD-induced murine diabetes

**Parameters**	**ND**	**DM**
n	7	19
**Blood glucose (mmol/mol)**	8.5±0.2	22.9±0.5****
**HbA1c (%)**	3.8±0.2	6.9±0.5**
**Body Weight (g)**	33.3±0.9	33.3±0.9
**Fat mass (g)**	8.48±2.0	7.51±3.9
**Lean mass (g)**	26.7±1.9	26.7±1.9
**Glucose tolerance (AUC)**	2396±163	4654±128****
**Insulin tolerance (AUC)**	471±29	540±80

Physiological parameters measured to assess the degree of diabetes-mellitus (DM) in STZ-HFD treated mice at endpoint. Data presented as mean ± SEM and analysed using an unpaired t-test. Statistical significance was assumed at P <0.05, ** P < 0.01, **** P < 0.0001. AUC = area under the curve, DM = diabetes mellitus.

Supplementary Table 2 – Endpoint M-Mode echocardiography for assessing cardiac systolic function in murine diabetes

**Parameters**	**ND**	**DM**
n	7	19
**Heart rate (bpm)**	390±33	414±16
**AWd (mm)**	0.73±0.04	0.76±0.03
**LVEDD (mm)**	4.08±0.08	4.07±0.07
**PWd (mm)**	0.75±0.05	0.79±0.02
**LVESD (mm)**	2.86±0.06	2.6±0.08
**Fractional shortening (%)**	29.8±1.35	36.1±1.47*****

Data presented as mean ± SEM and analysed using an unpaired t-test. Statistical significance was assumed at P <0.05. AWd; anterior wall diastolic thickness, LVEDD; LV end diastolic dimension, PWd; posterior wall diastolic thickness, DM = diabetes mellitus.

Supplementary Table 3 – Organ weights

**Organ**	**ND**	**DM**
n	7	19
Tibial length (mm)	18.1±0.1	18.1±0.1
Heart weight (mg)	130.2±3.3	154.2±9.0
Liver weight (g)	1.5±0.05	2.1±1.1******
Spleen weight (mg)	99.5±3.6	121.7±8.5
Heart weight/tibial length	7.2±0.2	7.9±0.2
Liver weight/tibial length	84.7±2.7	107.7±3.8******
Spleen weight/tibial length	5.5±0.2	7.2±0.5

Represents the raw organ weights and weights relative to tibia length. Data are presented as mean ± SEM and analysed by unpaired t-test. * $P < 0.05$.

Supplementary Table 4 – Flow cytometry antibody panel utilised in whole blood from mice

Antibody Target/Dye	Cat #	Company	Clone	Staining pattern
DAPI	D9542-5MG	Sigma-Aldrich	N/A	Dead cells
CD45R/B220	103241	BD Biosciences	RA3-6B2	B-cells
CD3ε	11-0033-81	BD Biosciences	500A2	T-cells
Gr1 (Ly6G/Ly6C)	552093	BD Biosciences	RB6-8C5	Monocytes & Neutrophils
CD115	135506	BD Biosciences	AFS98	Monocytes
CD4	116016	BD Biosciences	RM4-4	$CD4^+$ T-cells
CD8	126612	BD Biosciences	YTS156.7.7	$CD8^+$ T-cells
CD45	557659	BD Biosciences	30-F11	Pan leukocytes

Lists the antibody cocktail utilised for whole blood flow cytometry. 7 antibodies and 1 dye (DAPI) were used in this study to detect a range of leukocytes. Note: cell types in column 5 are the identifiable cells for the purpose of this study and may not apply in different contexts. For more detailed information regarding flow cytometry gating strategies and cellular identification, see Supplementary Figure 3.

Supplementary Table 5 – Flow cytometry antibody panel utilised in myocardium from mice

Antibody Target/Dye	Cat #	Company	Clone	Staining pattern
CD31	740879	BD Biosciences	390	Endothelial cells
I-A/I-E (MHCII)	743876	BD Biosciences	2G9	B-cells, macrophages
CD11b	564443	BD Biosciences	M1/70	Myeloid cells
CD64 (a & b alloantigens)	740622	BD Biosciences	X54-5/7.1	Macrophages
CD146 (Mcam)	740827	BD Biosciences	ME-9F1	Mural cells
eBioscience™ Calcein Blue AM Viability Dye	65-0855-39	Invitrogen	N/A	Metabolically active cells
Ly6C	128012	Biolegend	HK1.4	Monocytes
CD59a	130-104-105	Miltenyi Biotec	REA287	Schwann cells
Ly6G	127648	Biolegend	1A8	Granulocytes
NK1.1	108716	Biolegend	PK136	Natural killer cells
CD39	143806	Biolegend	Duha59	Smooth muscle cells
SYTOX™ Green Dead Cell Stain	S34860	Invitrogen	N/A	Dead cells
CD90.2	105320	Invitrogen	30-H12	T-cells
CD45	557659	BD Biosciences	30-F11	Pan leukocytes

Lists the antibody cocktail utilised for flow cytometry of cardiac ventricles. 12 antibodies and 2 dyes (SYTOX green and Calcein blue) were used in this study to detect an array of cardiac non-myocytes. Note: cell types in column 5 are the identifiable cells for the purpose of this study and may not apply in different contexts. For more detailed information regarding flow cytometry gating strategies and cellular identification, see Supplementary Figure 2.

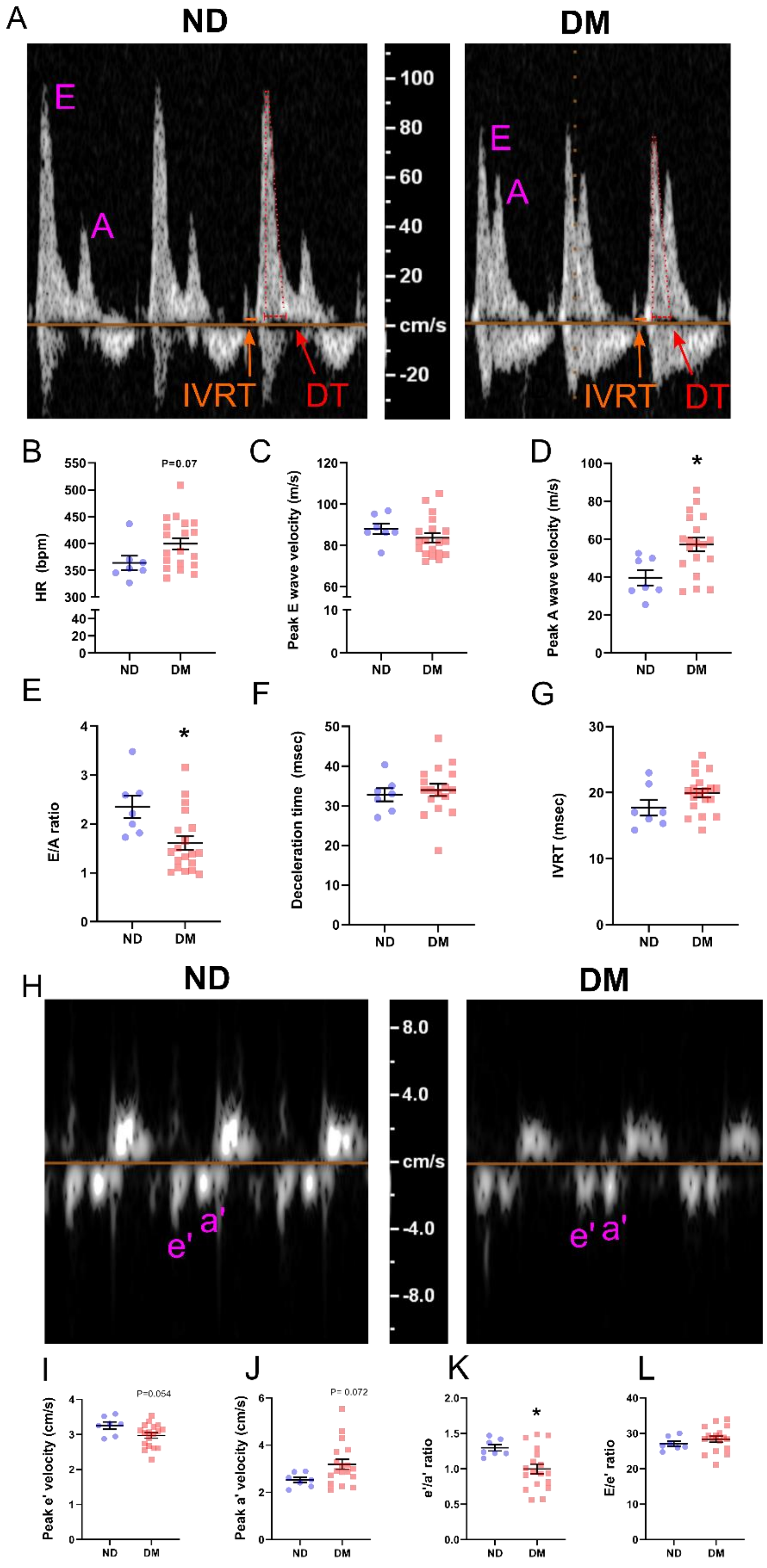

Supplementary Figure 1: (A) Representative images of transmitral annular blood flow via Doppler echocardiography. Quantified Doppler flow; **(B)** Anaesthetised heart rate (HR), **(C)** peak E-wave velocity, **(D)** A-wave velocity **(E)** E:A ratio, **(F)** deceleration time (DT) and **(G)** isovolumic relaxation time (IVRT). **(H)** Shows representative images for tissue Doppler echocardiography, quantified in figures I-L. **(I)** Peak e' velocity (P=0.054), **(J)** peak a' velocity (P = 0.072), **(K)** e':a' ratio, **(L)** E:e' ratio. ND = non-diabetic, DM = diabetes mellitus. Data presented as mean ± SEM and individual data points, and analysed using an unpaired t-test. Statistical significance was assumed at *P* <0.05.

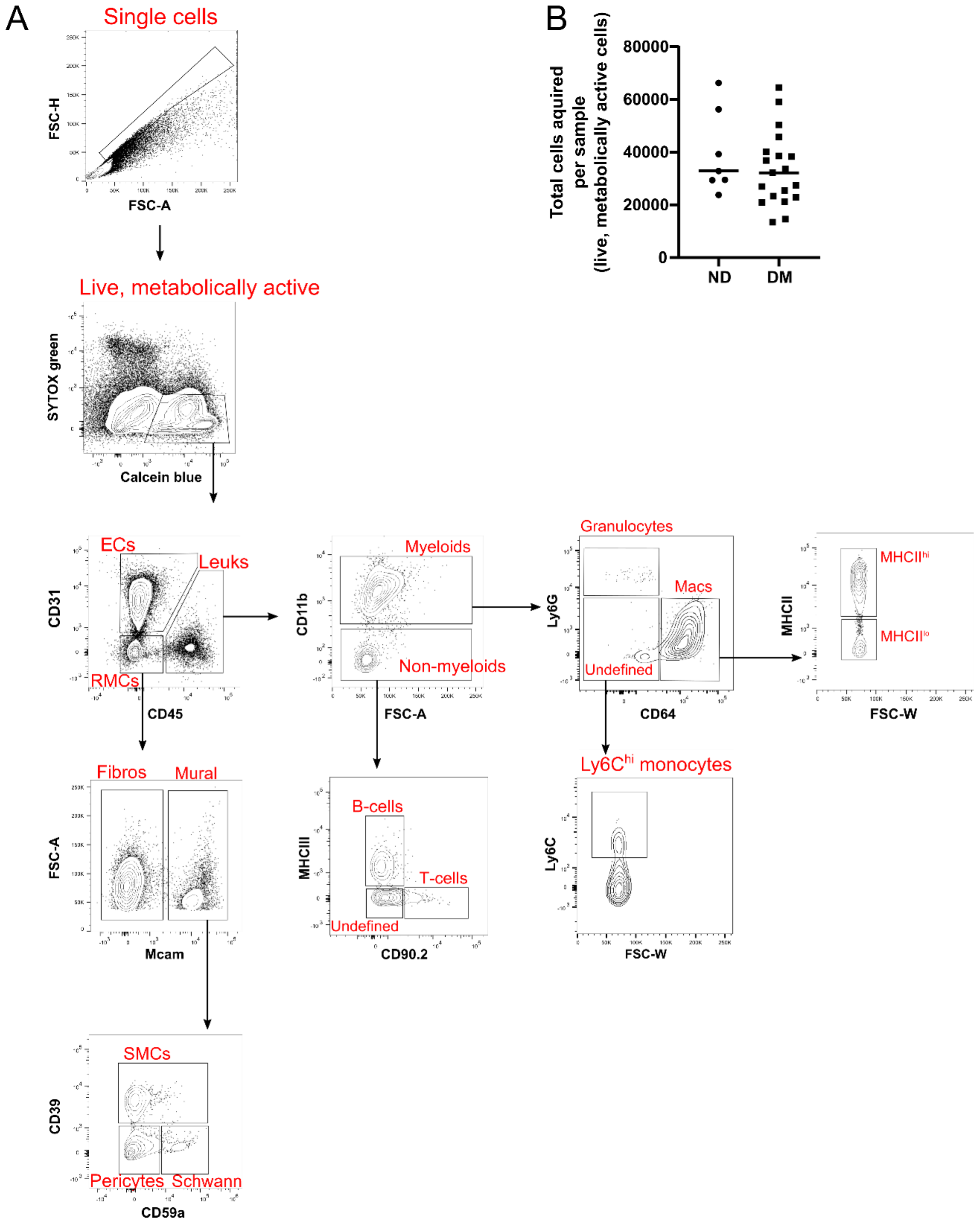

Supplementary Figure 2: Flow cytometry gating strategies – *Heart*

A) Illustrates the gating strategies used for identification of cardiac non-myocyte cell populations. Single, intact cells are first identified by the FSC-A/FSC-H gate. Next, cells are deemed 'live and metabolically active' by gating all SYTOX$^-$Calcein$^+$ events as indicated. Live, metabolically active cells were then identified based on their cell clustering to each respective antibody (listed on the x and y-axes). ECs = Endothelial cells, RMCs = Resident mesenchymal cells, Leuks = Leukocytes, Undefined = Undefined cells, Macs = Macrophages, MHCII$^{hi/lo}$ = MHCII$^{hi/lo}$ macrophages, Fibros = Fibroblasts, Mural = Mural cells, SMCs = Smooth Muscle Cells, Schwann = Schwann cells, FSC-A = forward scatter area, FSC-W = forward scatter width, FSC-H = forward scatter height. **B)** Depicts the total number of live, metabolically active cells acquired per sample (from flow cytometry), split by treatment (ND = non-diabetic, DM = diabetes mellitus. Data is presented as individual values. Each line indicates the median. *P* = NS (Student's unpaired *t*-test).

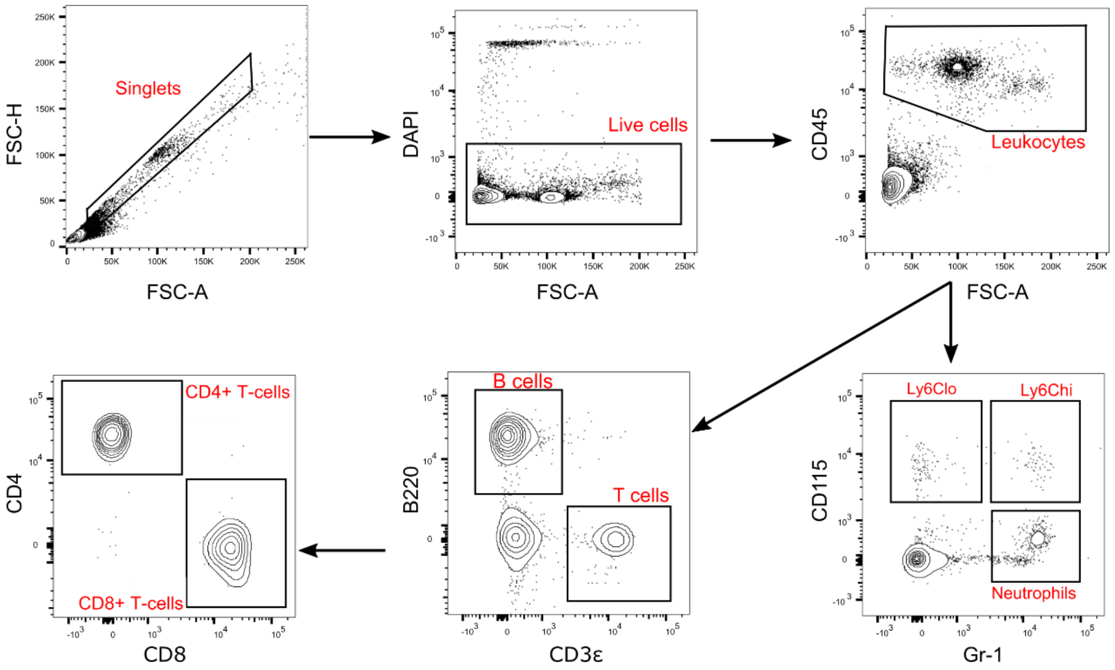

Supplementary Figure 3 – Flow cytometry gating strategies - *Blood*

Illustrates the gating strategies used for identification of circulating leukocyte populations. Single, intact cells are first identified by the FSC-A/FSC-H gate. Live cells were identified as DAPI$^-$ (4′,6-diamidino-2-phenylindole), after which cells are assigned as described in Supplementary Figure 2. FSC-A = forward scatter area, FSC-W = forward scatter width, FSC-H = forward scatter height.

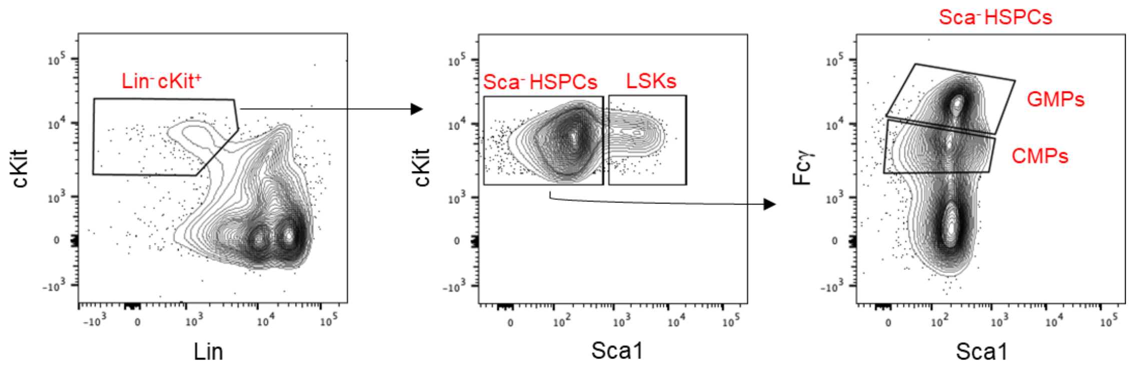

Supplementary Figure 4 –Flow cytometry gating strategies – *Bone marrow*

Illustrates the gating strategies used for identification of bone marrow progenitors. Lin$^-$ = lineage negative, HSPC = haematopoietic stem cell, LSK = lineage$^-$ cKit$^+$ cells, FSC-H = forward scatter height. Lineage cocktail = CD3, CD19, CD2, B220, TER119, CD11b, Gr-1, CD8, CD4.

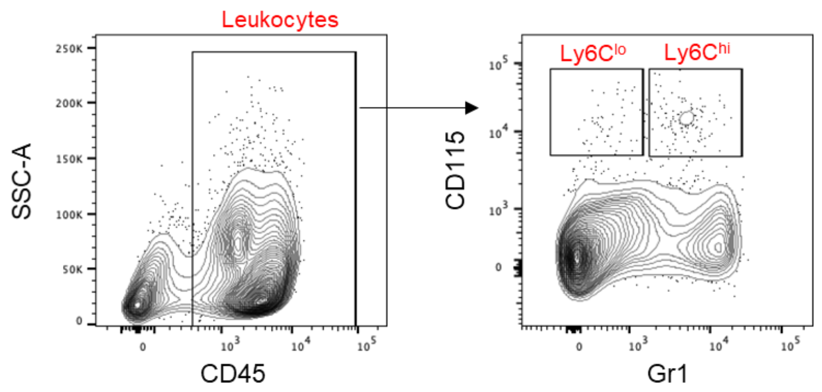

Supplementary Figure 5 – Flow cytometry gating strategies – ***Spleen***

Illustrates the gating strategies used for identification of splenic monocytes. SSC-A = side scatter area.

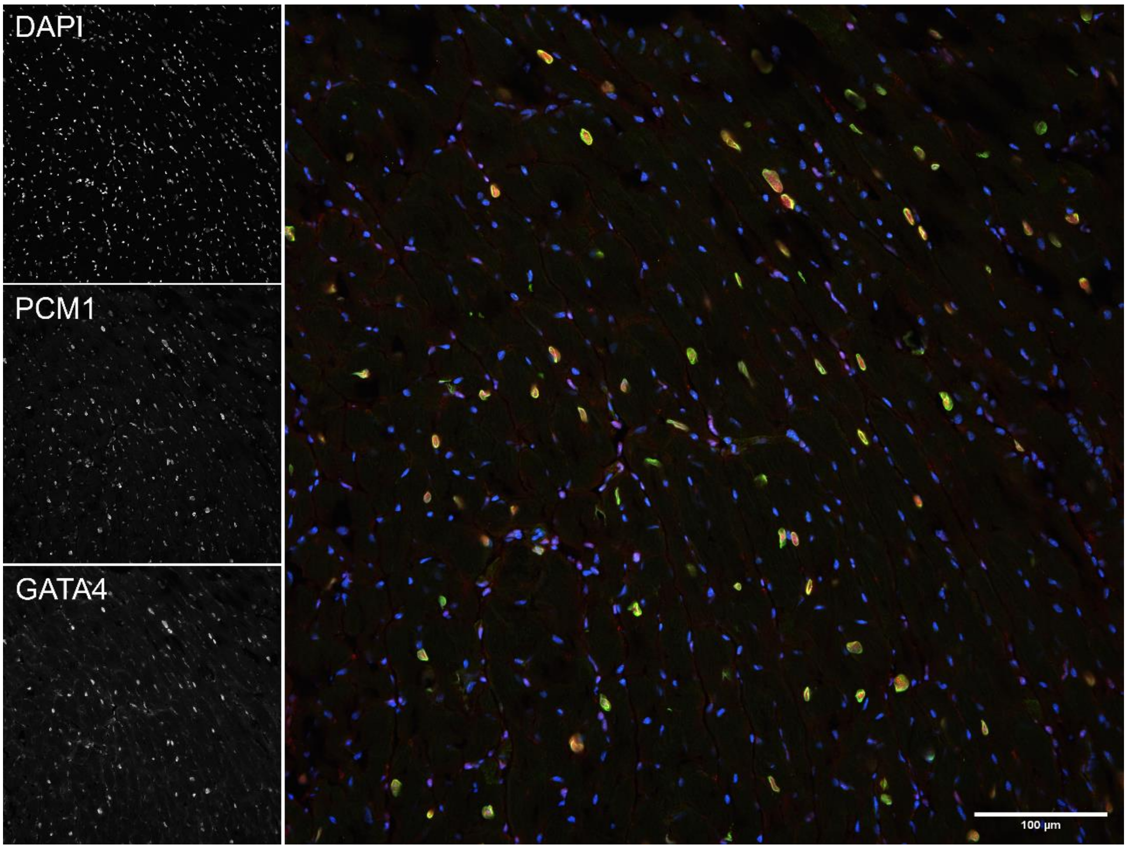

Supplementary Figure 6 – Histological identification of resident mesenchymal cells

Representative micrograph of murine left-ventricle stained with PCM1 and GATA4 antibodies, counter-stained with DAPI. Monochrome images (left) indicate the positive signals acquired for nuclei enumeration. Each channel is then merged and displayed in colour (right).

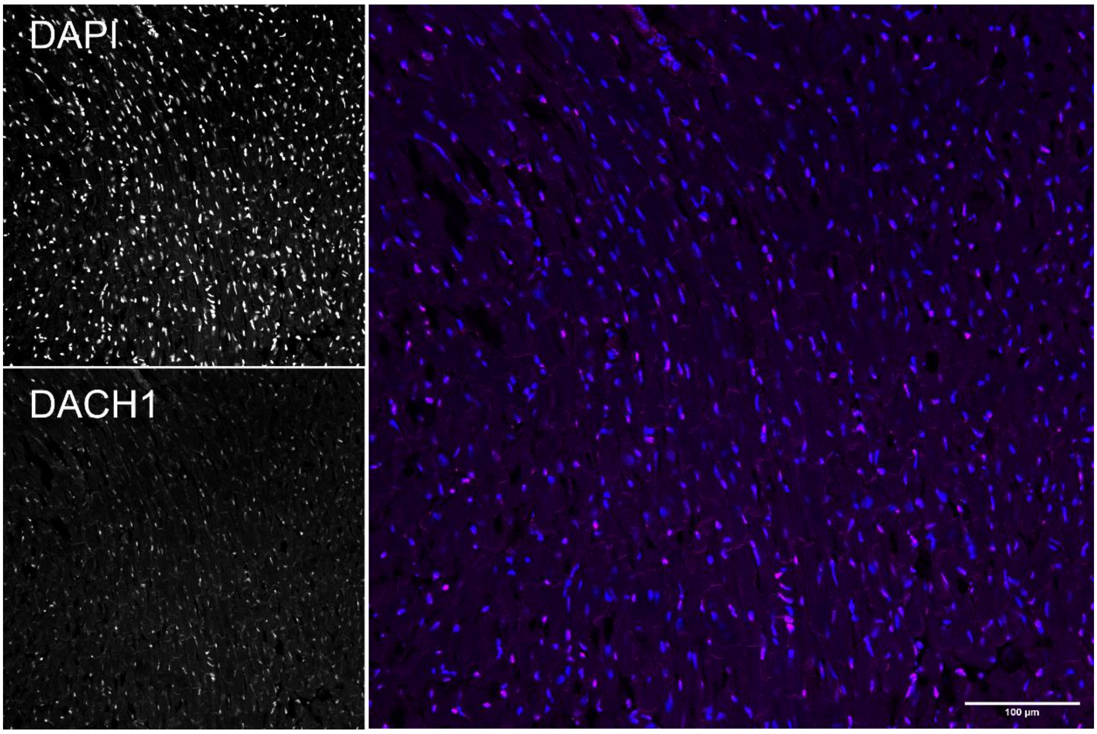

Supplementary Figure 7 – Histological identification of endothelial cells

Representative micrograph of murine left-ventricle stained with DACH1 and counter-stained with DAPI. Monochrome images (left) indicate the positive signals acquired for nuclei enumeration. Both channels are then merged and displayed in colour (right).

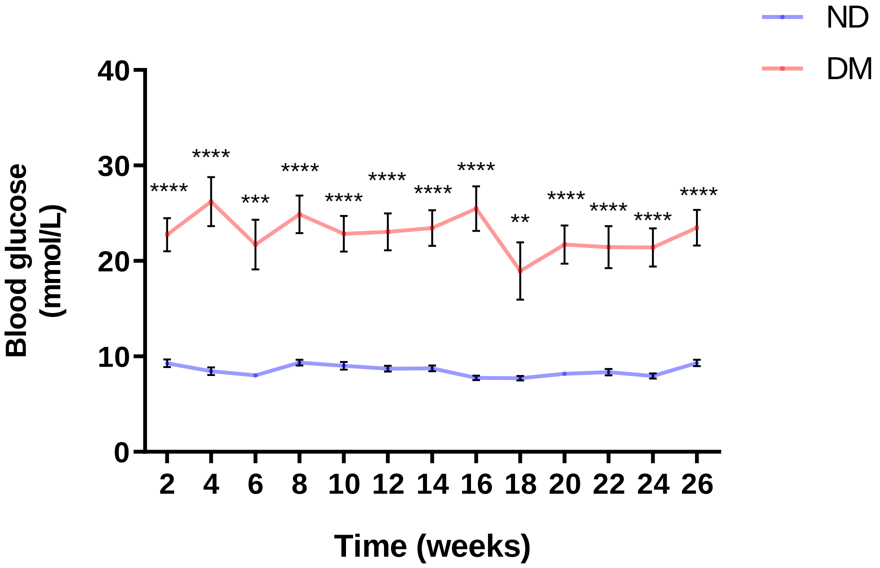

Supplementary Figure 8 – Chronic hyperglycaemia is evident in diabetic mice throughout study duration

Hyperglycaemia was first detected 2-weeks after the commencement of STZ-HFD administration, and remains elevated until endpoint (measured fortnightly). Data is presented as mean ± SEM. Statistical significance was determined by a repeated measures ANOVA using a Tukey's multiple comparison post-hoc test. $^{**}P < 0.01$, $^{***}P < 0.001$, $^{****}P < 0.0001$.
